# Supplementary material for: Double strand breaks drive toxicity in Huntington’s disease mice with or without somatic expansion
Source: bioRxiv. 2025 May 28:2025.05.27.654663. Preprint. [Version 1] doi: 10.1101/2025.05.27.654663 (PMC12154654; doi:10.1101/2025.05.27.654663)
Supplement: 1 [file NIHPP2025.05.27.654663V1-supplement-1.pdf]

bioRxiv preprint doi: https://doi.org/10.1101/2025.05.27.654663; this version posted May 28, 2025. The copyright holder for this preprint (which was not certified by peer review) is the author/funder. This article is a US Government work. It is not subject to copyright under 17 USC 105 and is also made available for use under a CC0 license.

## SUPPLEMENTAL FIGURE LEGENDS

**Supplemental Fig. 1: Somatic expansion occurs in the brains of *HdhQ(150/150*) mice and is longest in the STR.**

(A) The DNA was purified from the tails or tissue of animals was checked for quality by PCR. Good quality DNA was sent to TransnetYX CAG sizing by for high-resolution capillary electrophoreses. Representative examples of Genescan for somatic expansion in pairs showing the tail at 4wks and in the cortical brain tissue at 30 weeks in four *HdhQ(150/150*) littermates, IDs: 668, 621, 162, and 163. The dotted line indicates the midpoint of the tail a at 4wks, taken as the size of the inherited allele.

**Supplemental Fig. 2: MSH3 and MSH6 expression is prominent in neurons and glia of WT and *HdhQ(150/150*) mice**. (A) SDS-PAGE results for MSH2, MSH3 and MSH6 corresponding to the plots in Fig. 1. Details are described in the Fig. 1 legend. Protein extracts were resolved in six technical replicate SDS-PAGE gels; three gels for proteins measured in 10 wk animals (left) and three for proteins measured in 75 wk animals (right). Two siblings are shown side by side in each gel and are numbered as 1 and 2. Each blot was probed with indicated antibodies to a representative protein (P) or GAPDH (C). The protein expression data are normalized relative to the WT CBL. Error bars represent minimum (lower bar) and maximum (upper bar) values for the clonal samples at each age and were similar. The protein antibodies are listed in Supplementary Table 1. (B,C) Additional examples of magnified images of MSH3 as detected by MSH3 antibody IF staining (green) as shown in Fig, 2. Cell images were obtained from tissue sections from the STR of WT or HD animals of 70-90 wk. Neurons were NeuN (+) and GL were NeuN(-), as in Fig. 2. MSH3 expression is low in GL compared to neurons. The blue outline in the GL panels indicate the position of the nucleus as defined by DAPI staining. Scale bar is 10µm. The protein antibodies for MSH2, MSH3 and MSH6 are listed in Supplementary Table 2.

bioRxiv preprint doi: https://doi.org/10.1101/2025.05.27.654663; this version posted May 28, 2025. The copyright holder for this preprint (which was not certified by peer review) is the author/funder. This article is a US Government work. It is not subject to copyright under 17 USC 105 and is also made available for use under a CC0 license.

**Supplemental Fig. 3. WT and *HdhQ(150/150)* mice express the machinery to carry out DNA repair.**

(A) SDS-PAGE results corresponding to the plots for DNA repair proteins in multiple pathways shown in Fig. 3. The representative pathway proteins measured in include Apurinic/apyrimidinic (AP) endonuclease (APE1); Xeroderma Pigmentosum Group A protein (XPA), Xeroderma Pigmentosum Group F protein (XPF), and Excision Repair Cross-complementation group 1 (ERCC1); Meiotic Recombination 11 Homolog 1 (MRE11); X-ray repair cross-complementing 6 (Ku70); X-ray repair cross-complementing 5 (Ku80). The proteins extracts were resolved in ten technical replicate SDS-PAGE gels; five gels for proteins measured in 10 wk animals (left) and five for proteins measured in 75 wk animals (right). Two siblings are shown side by side in each gel and are numbered as 1 and 2. Each blot was probed with indicated antibodies to a representative pathway protein (P) or GAPDH (C). The protein expression data are normalized relative to the WT CBL. Error bars represent minimum (lower bar) and maximum (upper bar) values for the clonal samples at each age and were similar. Source data and uncropped gels are provided in Supplementary Source File. The protein antibodies are listed in Supplementary Table 2.

**Supplemental Fig. 4: Htt and mhtt interacts with Ku70, Ku80 and RPA1-3 in the STR of *HdhQ(150/150)* mice**

(A) Schematic diagram for immunoprecipitation and mass spectrometry (IP-MS) analyses of htt/Mhtt interactions with DNA repair proteins in the STR of WT or *HdhQ(150/150)* mice. The extracts from the STR of n=6 mice were immunoprecipitated using specific antibodies for htt and subjected to MS analysis to identify the binding partners. (B) Results for MS analysis of (A) for the pulldown products with the htt antibody for DNA repair proteins in the STR of WT (green) or *HdhQ(150/150)* (red) mice. Protein detected are Ku 70, Ku80 and RPA single strand binding proteins (right), which were like the MS results obtained in NIH3T293 cells (Fig. 4C,D). (C) No IP

bioRxiv preprint doi: https://doi.org/10.1101/2025.05.27.654663; this version posted May 28, 2025. The copyright holder for this preprint (which was not certified by peer review) is the author/funder. This article is a US Government work. It is not subject to copyright under 17 USC 105 and is also made available for use under a CC0 license.

products were collected using a nonspecific antibody (control, blue). MS analysis was performed by the Mass spectrometry Facility at University of California, Davis (UC Davis).

**Supplemental Fig. 5. Radiation induced damage does not kill brain cells.**

(A,B) Purified glia were isolated from the CBL (A) or STR (B) of WT or *HdhQ(150/150)* animals and exposed to 2Gy radiation. Shown are images for n=5 random fields taken 24 hrs post irradiation. Scale bar is 10µm. The cells are intact and show good morphology. (C) Quantification of cell number in 10-20 random fields among n=3 platings. Cell counts were determined using a hemocytometer and the number was plotted for the STR and CBL from each genotype, WT (light gray) and HD (dark gray). There was no statistical difference among samples.

**Supplemental Fig. 6: DSBs accumulate in neurons and Glia of WT and *HdhQ(150/150)* animals**

Additional examples of images for DSBs measurements in tissue sections from the STR of 70-90wk WT (left) or *HdhQ(150/150)*(right) animals, as described in Fig, 6 of main text. GL indicates glia and Neu indicates neurons. (Panel 1) An example of a random tissue field from the sections used for staining. The tissue sections were co-stained with nuclear DAPI (blue), neuronal NeuN (green) and DSBs marker γH2AX (red. Images shown are overlays of all three. (Panels 2-5) Individual magnified images of neurons and glia (GL) from the tissue sections. Scale bar is 5µm.

**Supplemental Fig. 7: No loss of neurons in *zQ17 or zQ175/MSH3(-/-)* animals by 6 months.**

(A) (Left) Tissue section representation showing the position of the STR (red) and the tissue used for IF measurements (yellow box); (Right) images of tissue sections from the WT and *zQ175* mice at 6 months of age, as indicated. (B) Magnified images of tissue sections in (A) stained with an antibody to the neuronal marker, NeuN. Scale bar is 50µm. (C) Quantification of neuronal counts in tissue section from the STR of WT (left, white box) and zQ175 (right, gray box) animals at 6

bioRxiv preprint doi: https://doi.org/10.1101/2025.05.27.654663; this version posted May 28, 2025. The copyright holder for this preprint (which was not certified by peer review) is the author/funder. This article is a US Government work. It is not subject to copyright under 17 USC 105 and is also made available for use under a CC0 license.

months. The whole STR was imaged using ImageJ and the number of neurons per unit area was determined from the NeuN staining intensity using the software. The NeuN intensity was divided by the tile area to generate neuron density. Data taken from n=5 random fields from n=3 tissue slices from the STR of n=3 animals per genotype (WT vs HD). (ns) No statistical differences were observed in the neuronal counts.

**Supplemental Fig. 8: XJB-5-131 treatment of *HdhQ(150/150*) mice suppresses neuronal pathology with minimal effects on somatic expansion**. The Genecan analysis of CAG repeat tracts of saline vehicle (Vh) or XJB-5-131 treatment at 90wks (60wks aging + 30 weeks treatment). Dotted line indicates CAG tract in tail at birth.

bioRxiv preprint doi: https://doi.org/10.1101/2025.05.27.654663; this version posted May 28, 2025. The copyright holder for this preprint (w
was not certified by peer review) is the author/funder. This article is a US Government work. It is not subject to copyright under 17 USC 1
and is also made available for use under a CC0 license.

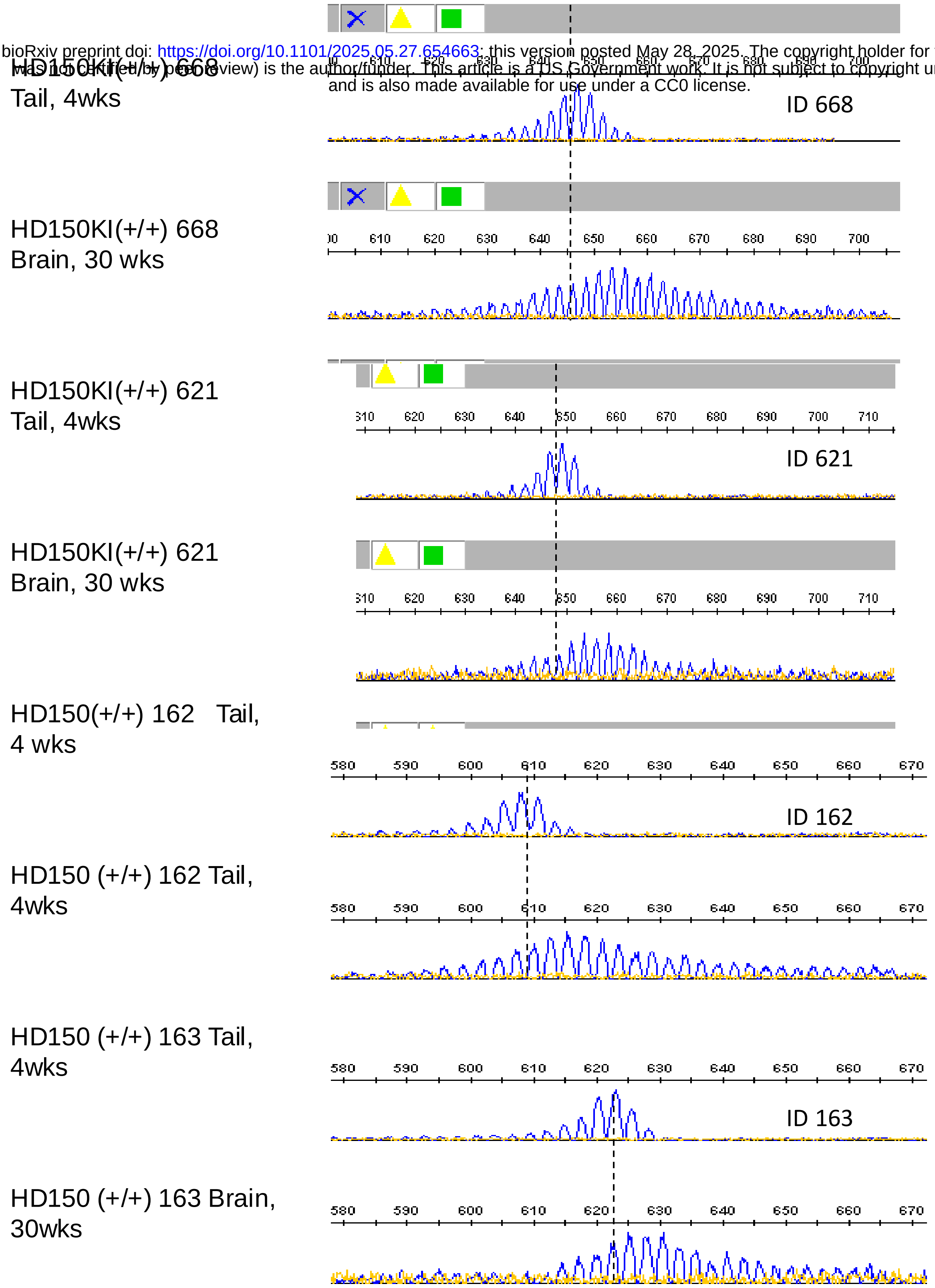


Supplemental Figure 1

bioRxiv preprint doi: https://doi.org/10.1101/2025.05.27.654663; this version posted May 28, 2025. The copyright holder for this preprint (w
was not certified by peer review) is the author/funder. This article is a US Government work. It is not subject to copyright under 17 USC 1
and is also made available for use under a CC0 license.

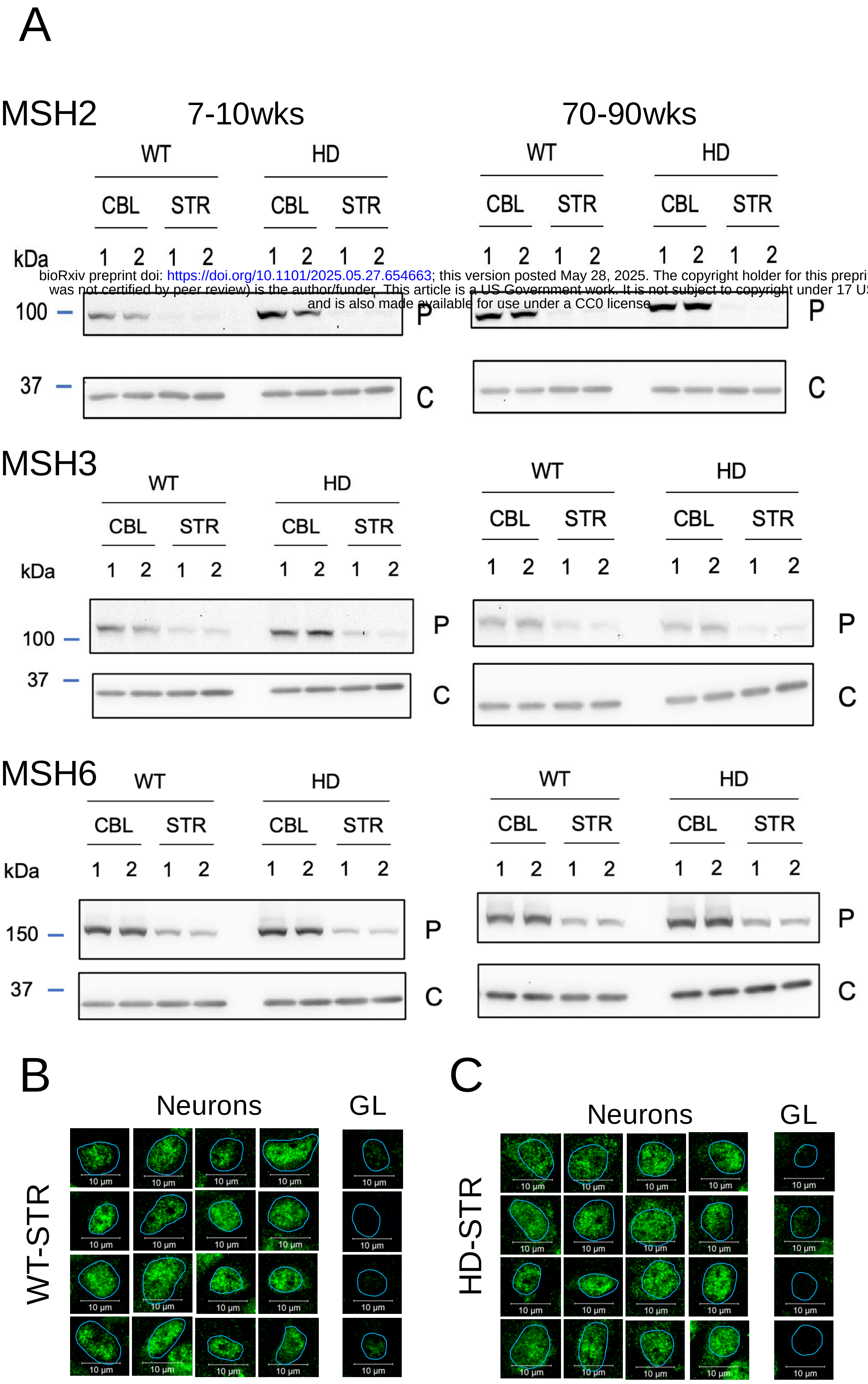


Supplemental Figure 2

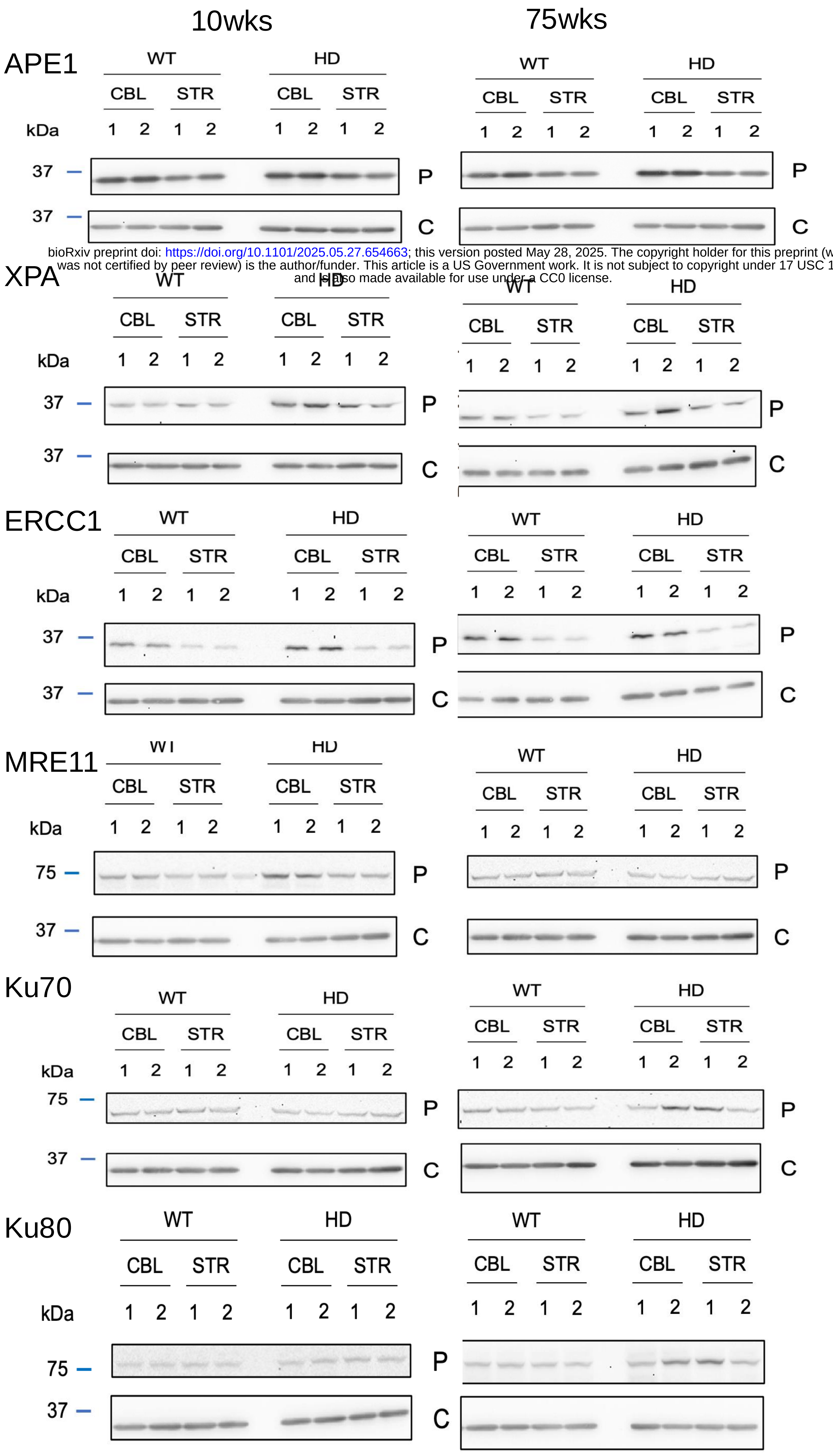


bioRxiv preprint doi: https://doi.org/10.1101/2025.05.27.654663; this version posted May 28, 2025. The copyright holder for this preprint (w
was not certified by peer review) is the author/funder. This article is a US Government work. It is not subject to copyright under 17 USC 1
and is also made available for use under a CC0 license.

Supplemental Figure 3

bioRxiv preprint doi: https://doi.org/10.1101/2025.05.27.654663; this version posted May 28, 2025. The copyright holder for this preprint (w
was not certified by peer review) is the author/funder. This article is a US Government work. It is not subject to copyright under 17 USC 1
and is also made available for use under a CC0 license.

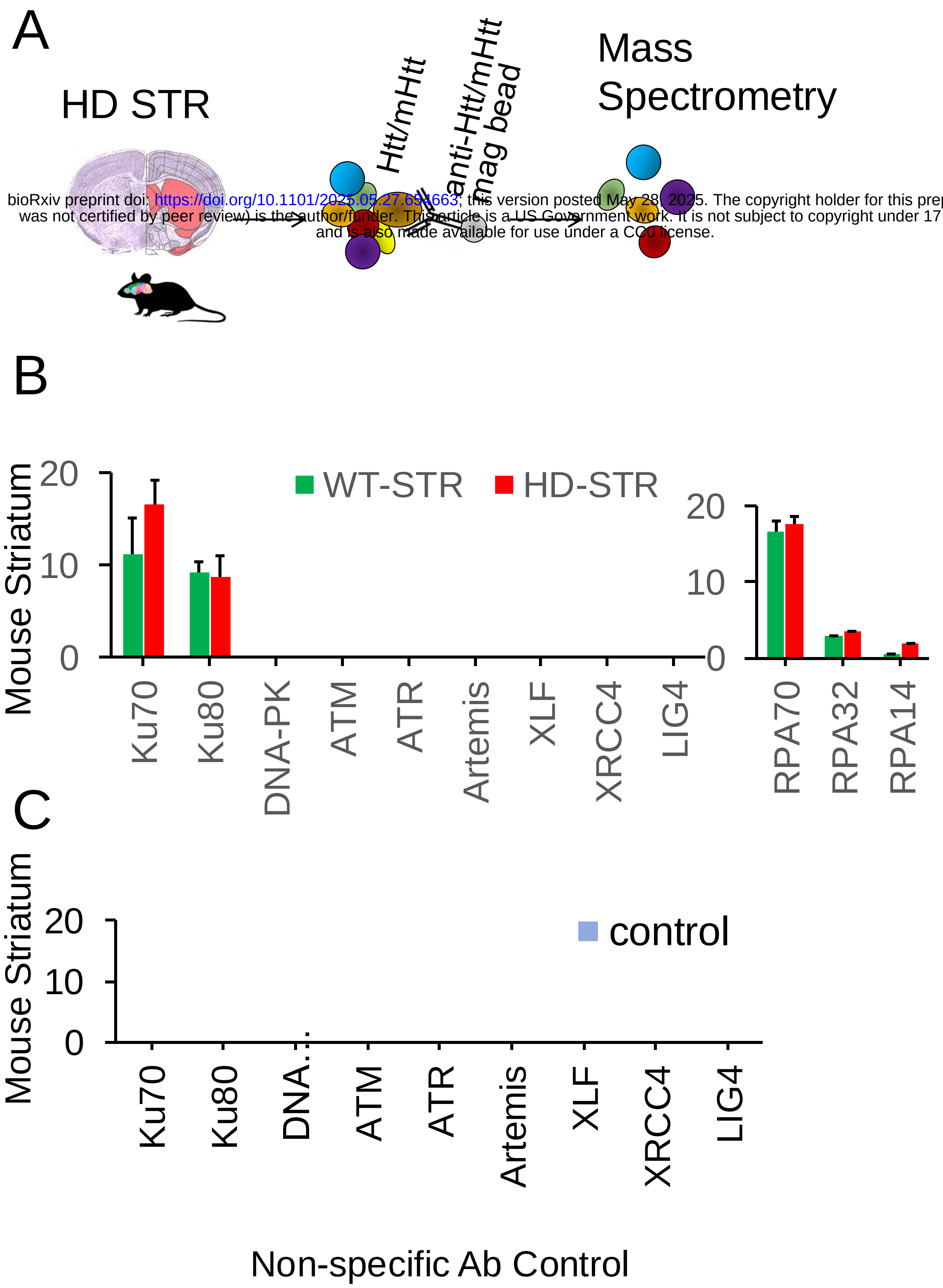


Supplemental Fig. 4

bioRxiv preprint doi: https://doi.org/10.1101/2025.05.27.654663; this version posted ... The copyright holder for this preprint (w... was not certified by peer review) ... this article is a US Government work. It is not subject to copyright under 17 USC 1... also made available for use under a CC0 license.

A

Glia form CBL (24hrs post irradiation)

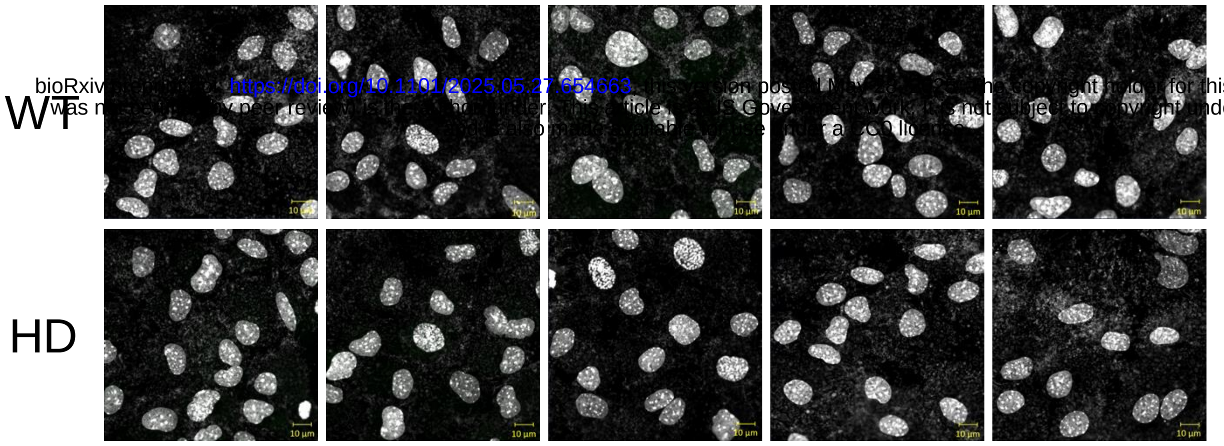


B

Glia from STR (24hrs post irradiation)

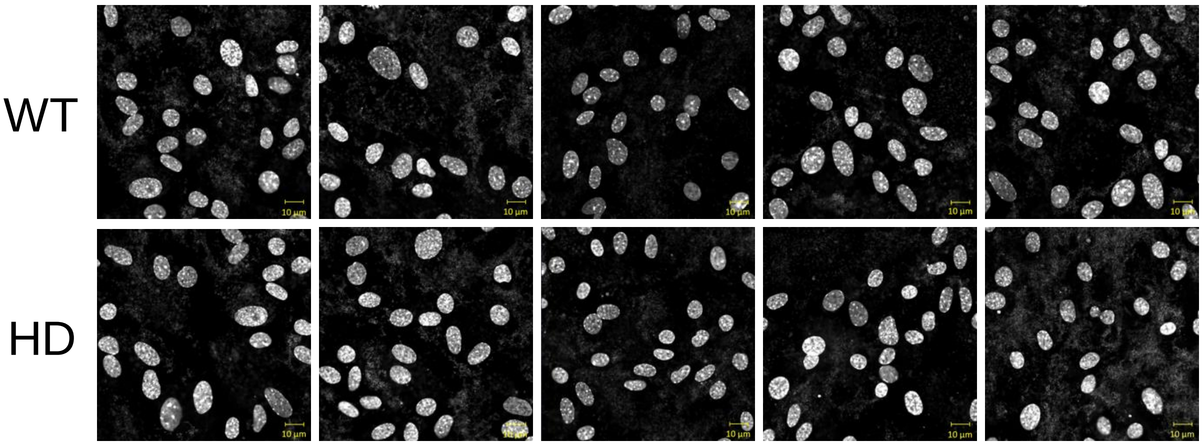


C

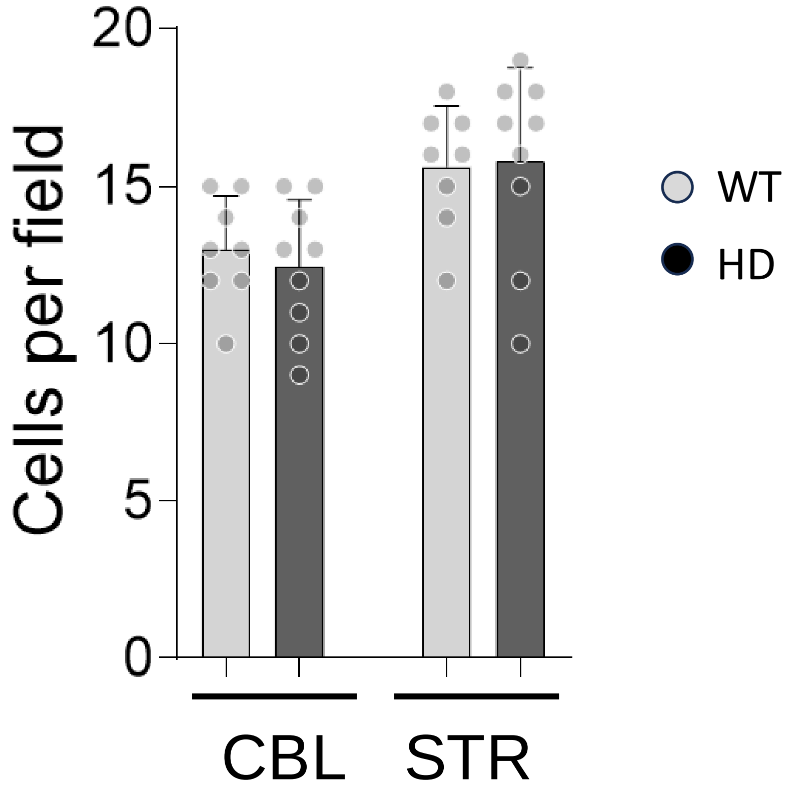


Supplemental Fig. 5

bioRxiv preprint doi: https://doi.org/10.1101/2025.05.27.654663; this version posted May 28, 2025. The copyright holder for this preprint (w
was not certified by peer review) is the author/funder. This article is a US Government work. It is not subject to copyright under 17 USC 1
and is also made available for use under a CC0 license.

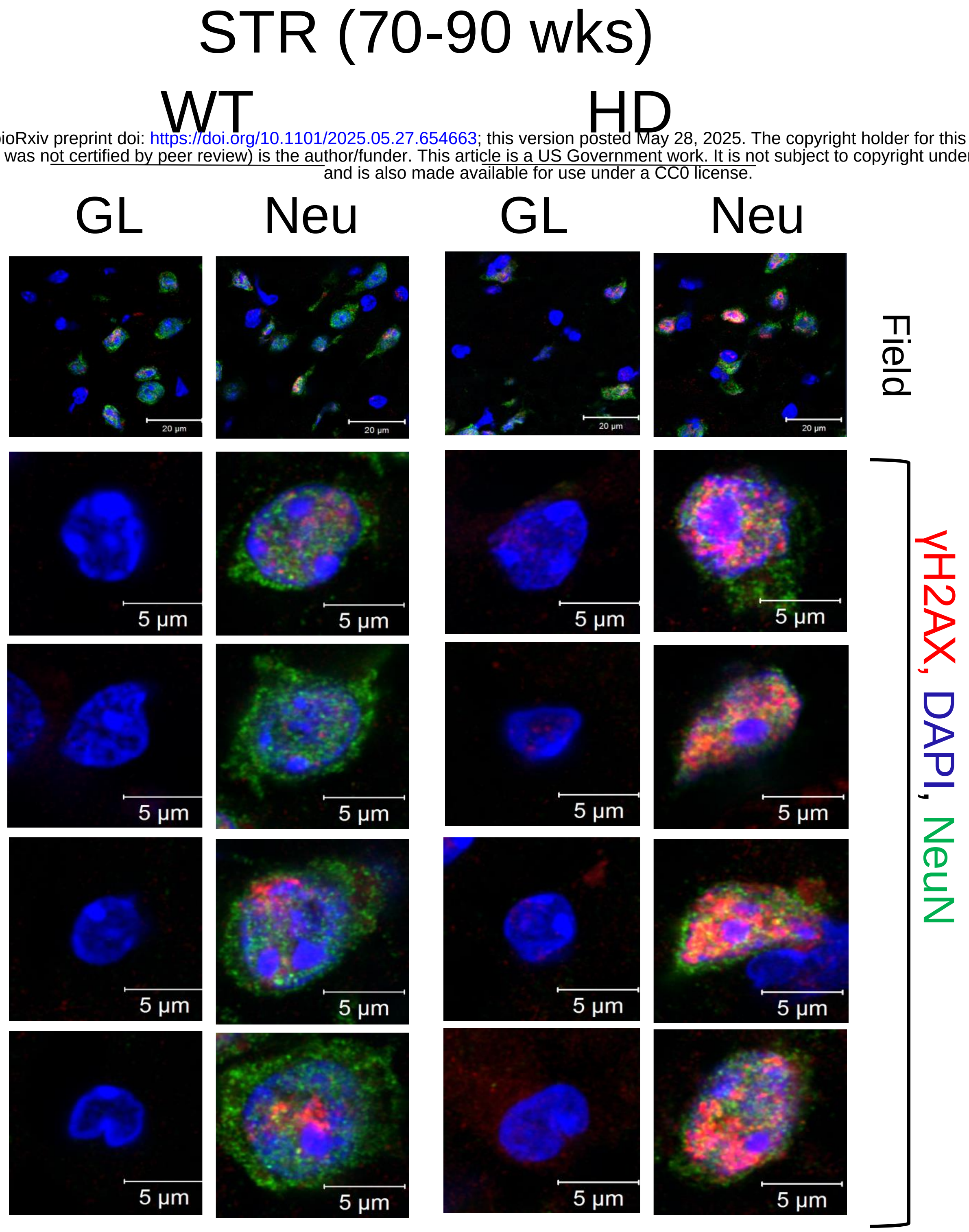


Supplemental Figure 6

bioRxiv preprint doi: https://doi.org/10.1101/2025.05.27.654663; this version posted May 28, 2025. The copyright holder for this preprint (w
was not certified by peer review) is the author/funder. This article is a US Government work. It is not subject to copyright under 17 USC 1
and is also made available for use under a CC0 license.

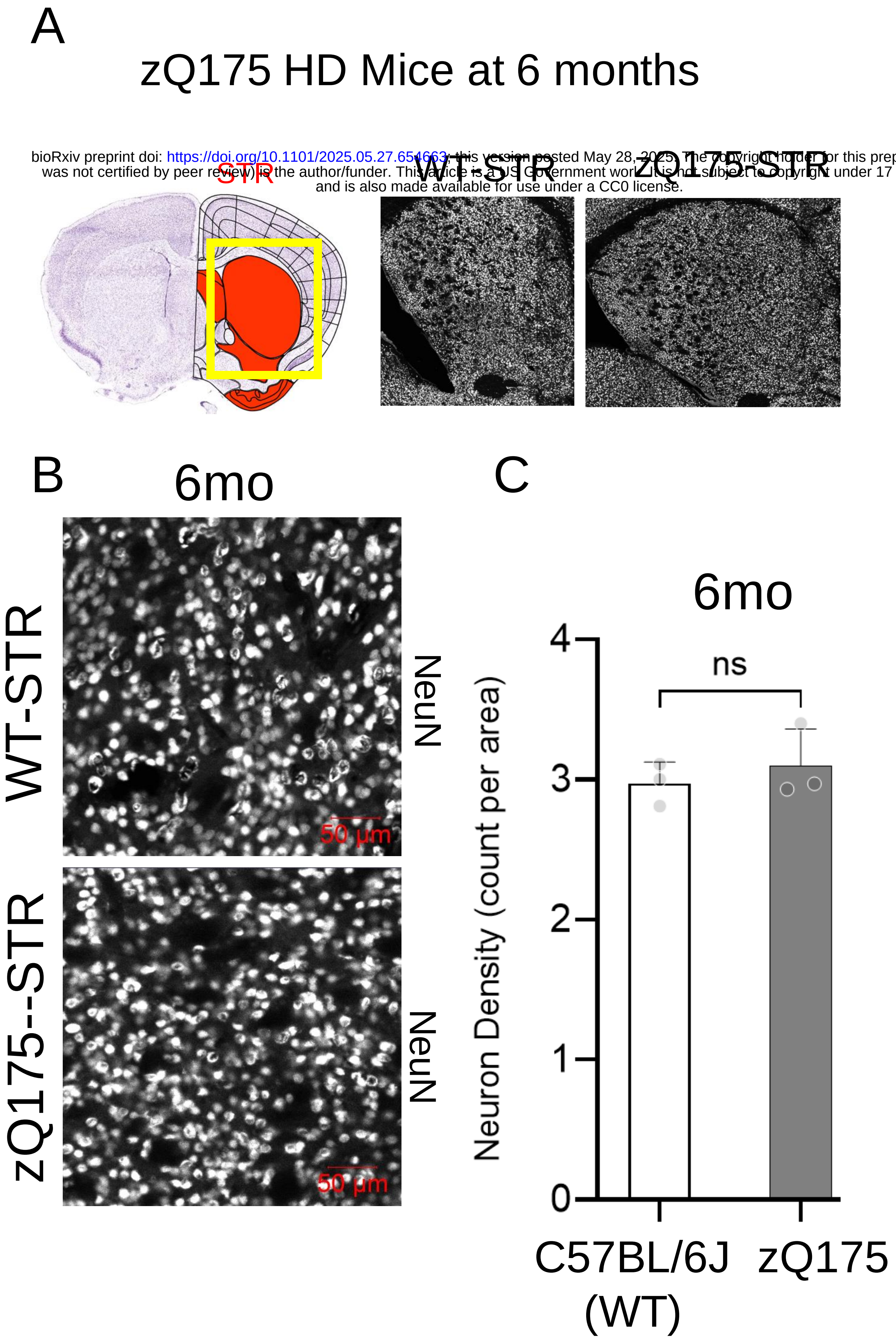


Supplemental Figure 7

bioRxiv preprint doi: https://doi.org/10.1101/2025.05.27.654663; this version posted May 28, 2025. The copyright holder for this preprint (w
was not certified by peer review) is the author/funder. This article is a US Government work. It is not subject to copyright under 17 USC 1
and is also made available for use under a CC0 license.

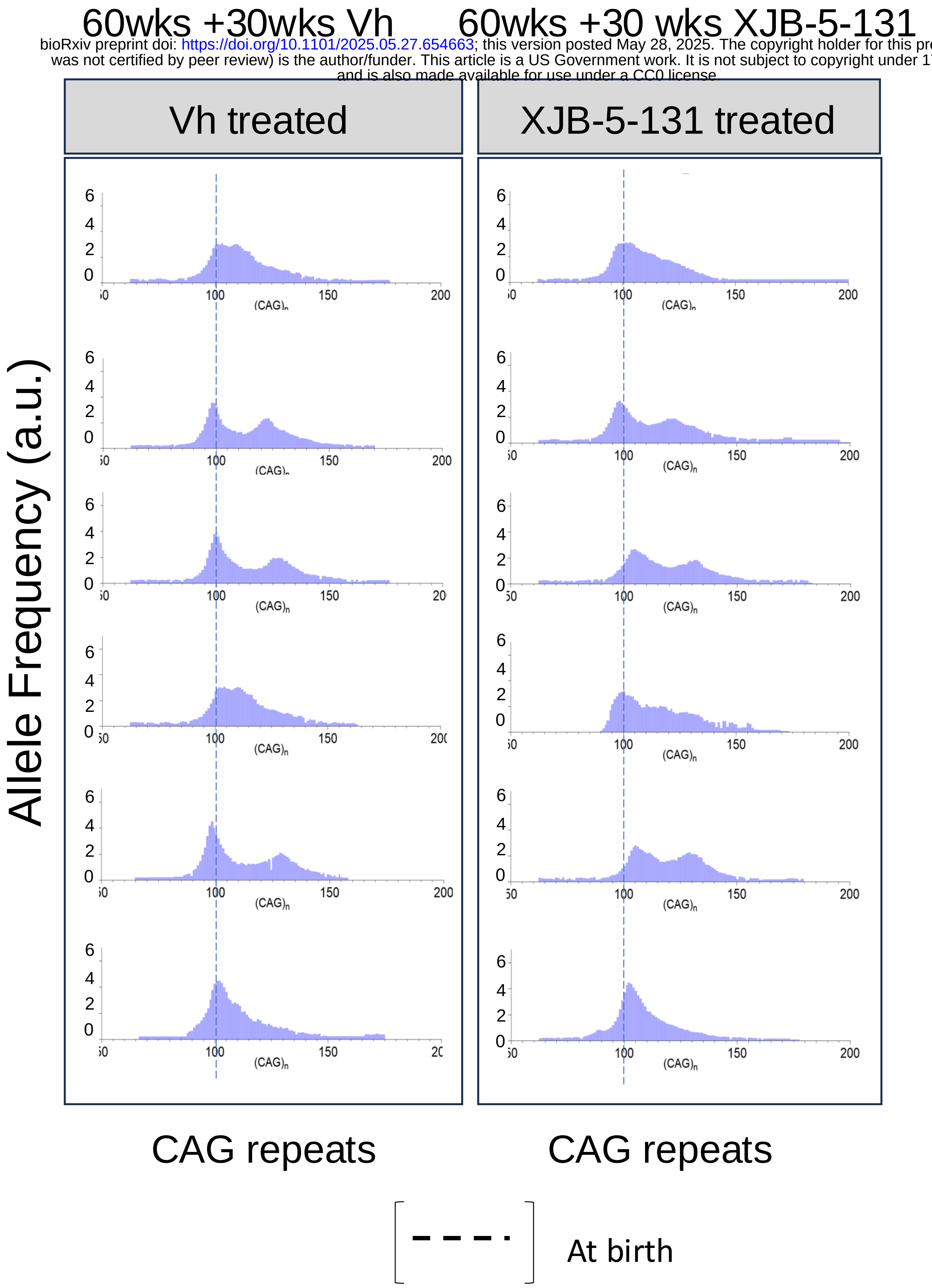


Supplemental Figure 8

bioRxiv preprint doi: https://doi.org/10.1101/2025.05.27.654663; this version posted May 28, 2025. The copyright holder for this preprint (which was not certified by peer review) is the author/funder. This article is a US Government work. It is not subject to copyright under 17 USC 105 and is also made available for use under a CC0 license.

## Supplementary Table 1: Key Resources.

| **Reagent or Resource** | | **Source** | **Catalogue ID** |
|---|---|---|---|
| **Antibody** | | | |
| Mouse anti-NeuN alexafluor-488 conjugate | 1:500 | EMD Millipore | MAB377X |
| Rabbit anti-NeuN alexafluor-647 conjugate | 1 :1,000 | Abcam | 190565 |
| Mouse anti-GFAP Cy3 conjugate | 1:500 | Abcam | ab49874 |
| Mouse anti-APE1 | 1:500 | Novus | 13B8E5C2 |
| Mouse anti-Ku80 | | Santa Cruz | 515736 |
| Mouse anti-ERCC1 | | Santa Cruz | 17809 |
| Rabbit anti-MSH2 | | Abcam | ab92471 |
| Mouse anti-MSH3 | | EMD Millipore | MABE324 |
| Rabbit anti-MSH6 | | Abcam | Ab92471 |
| Rabbit anti-XPA | | AbClonal | A1626 |
| Rabbit anti-MRE11 | | Novus | NB100-142 |
| Mouse anti-yH2AX | 1:400 – 1:1,000 | ThermoFisher | MA1-2022 |
| Rabbit anti-53BP1 | 1:1,000 | Bethyl | A300-237A |
| Donkey anti-Mouse alexafluor-488 conjugate | | Jackson Immunores. | 715-545-150 |
| Goat anti-Mouse alexafluor-568 conjugate | | Invitrogen | A21124 |
| Donkey anti-Rabbit alexafluor-488 conjugate | | Jackson Immunores. | 711-545-152 |
| Goat anti-Rabbit alexafluor-555 conjugate | | Invitrogen | A32732 |
| Goat anti-Mouse alexafluor-488+ conjugate | 1:1,000 | Invitrogen | A48286 |
| Goat anti-Rabbit alexafluor-555+ conjugate | 1:1,000 | Invitrogen | A48283 |
| | | | |
| | | | |
| **Chemicals, peptides, and recombinant proteins** | | | |
| Fc Receptor Block | | Innovex | NB309 |
| Background Buster | | Innovex | NB306 |
| Tissue-Tek O.C.T. Compound | | Sakura | 4583 |
| TrueBlack | | Biotium | 23007 |
| Monarch RNase A | | New England Biolabs | T3018L |
| ImmuMount | | Epredia | 9990402 |
| Nuclease P1 | | New England Biolabs | M0660 |
| Quick Calf Alkaline Phosphatase | | New England Biolabs | M0525 |
| CMV-cDNA26Q | | constructed in house | |
| CMV-cDNA51Q | | constructed in house | |

bioRxiv preprint doi: https://doi.org/10.1101/2025.05.27.654663; this version posted May 28, 2025. The copyright holder for this preprint (which was not certified by peer review) is the author/funder. This article is a US Government work. It is not subject to copyright under 17 USC 105 and is also made available for use under a CC0 license.

| | | | |
|---|---|---|---|
| T-PER Tissue Protein Extraction Reagent | | Thermo Scientific | 78510 |
| Halt protease inhibitor cocktail | | Thermo Scientific | 78420 |
| Pierce 660 Protein Assay reagent | | Thermo Scientific | 22660 |
| NuPAGE Sample Reducing Agent | | Invitrogen | NP0009 |
| Novex WedgeWell | | Thermo Fisher | XP04205BOX |
| Amersham ECL Western Blotting Reagent | | Sigma-Aldrich | GERPN2235 |
| CometAssay LMAgarose | | R&D Systems | 4250-050-02 |
| CometAssay Lysis Solution | | R&D Systems | 4250-050-01 |
| | | | |
| | | | |
| **Critical commercial assays** | | | |
| DNA/RNA Oxidation Assay | | Cayman chemicals | 589320 |
| DNeasy Blood and Tissue Kit | | Qiagen | 69504 |
| | | | |
| | | | |
| | | | |
| **Experimental models: Organisms/strains** | | | |
| *C57Bl/6J* male mice | | Jackson Labs | 000664 |
| NIH/3T3 fibroblasts | | ATCC | CRL-1658 |
| | | | |
| **Software and algorithms** | | | |
| ImageJ:Fiji | | imagej.net/software/fiji/ | Version 2.15.0 |
| Prism Graph Pad | | www.graphpad.com/features | version 9.5.1 |
| CometAssay Analysis Software | | R&D Systems | 4260-000-CS |
| VersaDoc MP 4000 Imaging System | | Biorad | Quantity One 1-D |
| Image Lab software | | Biorad | V1 |
| ZEN Black | | Zeiss | 2.1 SP3 FP3 |

## Supplementary Tabel 2. Antibody testing

| DNA Repair Pathway | Protein | WB Tried? | Antibodies Tried (Species) | Vendor | Catalog Number | Worked? (Mouse brain tissue lysates) | Worked? (Mouse astrocyte P1 culture lysates) |
|---|---|---|---|---|---|---|---|
| Homologous Recombination (HR) | BRCA1 | Yes | Ms | Novus Biologicals | MAB22101 | | No |
| | CtIP | Yes | Ms | Active Motif | 61141 | No | No |
| | BRCA2 | No | | | | | |
| Non-homologous End Joining (NHEJ) | Ku70 | Yes | Ms | Invitrogen | MA5-13110 | No | No |
| | | | Ms | SCBT | sc-17789 | No | |
| | | | Rb | CST | 4588S | | Yes |
| | Ku80 | Yes | Rb | Thermo Fisher | PA5-17454 | Not sure; many bands detected | |
| | | | Ms | SCBT | sc-515736 | Yes | Yes |
| | DNA-PK | Yes | Rb | Abcam | ab32566 | Yes, but band around 150 kDa | Yes, but smaller band around 150 kDa also detected |
| | Artemis | Yes | Ms | Bethyl Laboratories | A304-902A-M | Not sure; many bands detected | Yes, but band appears near 50 kDa |
| Base Excision Repair (BER) | APE1 | Yes | Ms | Novus Biologicals | 13B8E5C2 | Yes | |
| | OGG1 | Yes | Rb | Novus Biologicals | NB100-106 | Yes, but lots of bands | |
| | PARP-1 | Yes | Ms | Bio-Rad | MCA1522G | No | |
| Nucleotide Excision Repair (NER) | ERCC1 | Yes | Ms | SCBT | sc-17809 | Yes | Yes |
| | XPF | Yes | Ms | SCBT | sc-136153 | No | No |
| | XPG | No | | | | | |
| Mismatch Repair (MMR) | MSH2 | Yes | Rb | Abcam | ab92473 | Yes | Yes |
| | MSH3 | Yes | Ms | BD Biosciences | 611390 | No | |
| | | | Rb | Thermo Fisher | PA5-75306 | No | |
| | | | Ms | EMD Millipore | MABE324 | Yes | Yes |
| | MSH6 | Yes | Rb | Abcam | ab92471 | Yes | Yes |
